# Neural Network-Based Self-Tuning PID Control for Underwater Vehicles

**DOI:** 10.3390/s16091429

**Published:** 2016-09-05

**Authors:** Rodrigo Hernández-Alvarado, Luis Govinda García-Valdovinos, Tomás Salgado-Jiménez, Alfonso Gómez-Espinosa, Fernando Fonseca-Navarro

**Affiliations:** 1 Energy Division, Center for Engineering and Industrial Development-CIDESI, Santiago de Queretaro, Queretaro 76125, Mexico; ggarcia@cidesi.edu.mx (L.G.G.-V.); tsalgado@cidesi.edu.mx (T.S.-J.); ffonseca@posgrado.cidesi.edu.mx (F.F.-N.); 2 Tecnologico de Monterrey, Campus Queretaro, Ave. Epigmenio González 500, Fracc. San Pablo, Santiago de Queretaro, Queretaro 76130, Mexico; agomeze@itesm.mx

**Keywords:** neural networks, auto-tuning PID, ROV control, disturbances

## Abstract

For decades, PID (Proportional + Integral + Derivative)-like controllers have been successfully used in academia and industry for many kinds of plants. This is thanks to its simplicity and suitable performance in linear or linearized plants, and under certain conditions, in nonlinear ones. A number of PID controller gains tuning approaches have been proposed in the literature in the last decades; most of them off-line techniques. However, in those cases wherein plants are subject to continuous parametric changes or external disturbances, online gains tuning is a desirable choice. This is the case of modular underwater ROVs (Remotely Operated Vehicles) where parameters (weight, buoyancy, added mass, among others) change according to the tool it is fitted with. In practice, some amount of time is dedicated to tune the PID gains of a ROV. Once the best set of gains has been achieved the ROV is ready to work. However, when the vehicle changes its tool or it is subject to ocean currents, its performance deteriorates since the fixed set of gains is no longer valid for the new conditions. Thus, an online PID gains tuning algorithm should be implemented to overcome this problem. In this paper, an auto-tune PID-like controller based on Neural Networks (NN) is proposed. The NN plays the role of automatically estimating the suitable set of PID gains that achieves stability of the system. The NN adjusts online the controller gains that attain the smaller position tracking error. Simulation results are given considering an underactuated 6 DOF (degrees of freedom) underwater ROV. Real time experiments on an underactuated mini ROV are conducted to show the effectiveness of the proposed scheme.

## 1. Introduction

Underwater Remotely Operated Vehicles (ROVs) have been widely used in many subsea tasks, ranging from inspection to repair of underwater structures related mainly to the power and oil industry. Very often, according to the task, the ROV is required to continuously change its operating tool and/or to pick up and release loads causing a change in behavior. That results as an inherent change in its weight, buoyancy and hydrodynamic forces; and as a consequence, a decrease in the position tracking performance. In addition, ROVs have to deal with the highly dynamical underwater environment represented in the form of ocean currents and waves in shallow water. With this in mind, when the dynamic characteristics of the system are time dependent or the operating conditions of the system vary, it is necessary to re-tune the gains to obtain the desired performance, resulting in time consumption. In this paper, a self-tuning algorithm based on Neural Networks (NN) is proposed to automatically tune the gains of a PID (Proportional + Integral + Derivative) controller. The optimal set of gains is computed online with less computation effort by using desired and actual state variables. The self-tuning mechanism will avoid time consuming manual tuning of the PID controller and promises better results by providing PID controller settings as the system dynamics or operating points change.

With this in mind, a mix of control and a smart system might offer an accurate tune of the control gains online. Even when the state of art yields different tuning techniques, it is common to find controls poorly tuned such that their performance is limited. Intelligent control techniques include fuzzy control, neural networks or a mix of them; they have been widely used to control underwater and nonlinear systems, such as in [1,2,3], and have become an accurate option, though these algorithms do require long periods of training and tuning.

Control schemes vary from tracking to dynamic positioning [4,5] where their main target is to estimate and compensate for the unknown forces of changing environments. Research [6,7,8,9,10,11,12,13,14] present systems with a mix of neural networks and fuzzy control in which the training and rules of behavior are based on the desired states. Their performance is described as accurate when uncertainty and perturbations take place while performing a trajectory. Although the training periods are extremely long, there are also combinations of PID controls and a smart system aimed to auto-tune the gains of different systems such as: sub-aquatic [15,16], non linear [17,18,19,20,21,22], and others: [23,24,25,26].

In this paper, an auto-tune PID-like controller based on an online Neural Networks (NN) is implemented on Remotely Operated Vehicles (ROVs); for trajectory tracking with unknown disturbances. Simulation results are given considering the non-linear hydrodynamics of ROV Kaxan; including disturbances of ocean currents. Real time experiments on an underactuated mini ROV are conducted to show the effectiveness of the proposed scheme. For the remaining sections of this paper in Section 2 the general system model of 6 DOF underwater vehicles is presented, Section 3 includes the effect of ocean currents, Section 4 presents the Self-tuning Neural Network for PID Control, Section 5 describes the simulation results, and the experimental results are presented in Section 6; Finally in Section 7 the concluding remarks are provided.

## 2. General System Model of 6 DOF Underwater Vehicles

**Figure 1:**
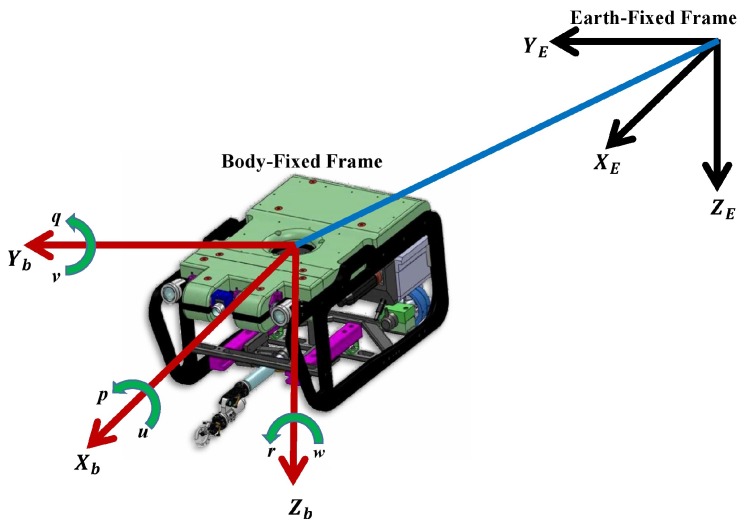
Frame coordinates of an underwater vehicle.

In [27], the nonlinear model of a 6 DOF to build the mathematical model that represents the underwater vehicle dynamics two reference frames were used; one referenced to earth (called the Earth-fixed frame) and another referenced to the vehicle (called the body-fixed frame), Figure 1.

### 2.1. Kinematic Model

The general velocity vector is represented as:

(1) $$\nu ={\left[\begin{array}{cc} \nu_{1} & \nu_{2} \end{array}\right]}^{T}={\left[\begin{array}{cccccc} u & v & w & p & q & r \end{array}\right]}^{T}$$

where u,v and *w* are components of the linear velocity in surge, sway and heave directions, respectively, and p,q and *r* are components of the angular velocity in roll, pitch and yaw, respectively.

The position vector $\eta_{1}\in R^{3}$ and orientation vector $\eta_{2}\in R^{3}$ coordinates expressed in the Earth-fixed frame are:

(2) $$\eta ={\left[\begin{array}{cc} \eta_{1} & \eta_{2} \end{array}\right]}^{T}={\left[\begin{array}{cccccc} x & y & z & \phi & \theta & \psi \end{array}\right]}^{T}$$

where *x*, *y* and *z* represent the Cartesian position in the Earth-fixed frame and *φ* represents the roll angle, *θ* the pitch angle and *ψ* the yaw angle.

The relationship between velocities on the fixed and Equations are [27,28].

(3) $$\left[\begin{array}{c} {\dot{\eta }}_{1}\\ {\dot{\eta }}_{2} \end{array}\right]=\left[\begin{array}{cc} J_{1}\left(\eta_{2}\right) & O_{3\times 3}\\ O_{3\times 3} & J_{2}\left(\eta_{2}\right) \end{array}\right]\left[\begin{array}{c} \nu_{1}\\ \nu_{2} \end{array}\right]$$

where $J_{1}\left(\eta_{2}\right)\in R^{3\times 3}$ is the rotation matrix which expresses the transformation from body to fixed frame, and $J_{2}\left(\eta_{2}\right)\in R^{3\times 3}$ is another transformation matrix that relates the angular velocity $\nu_{2}\in R^{3}$ with the time derivative of $\eta_{2}\in R^{3}$.

### 2.2. Hydrodynamic Model

Equations of motion expressed on the Equation [27],

(4) $$M\dot{\nu }+C\left(\nu \right)\nu +D\left(\nu \right)\nu +G\eta )=\tau$$

$$\dot{\eta }=J\left(\eta \right)\nu$$

where $\nu \in R^{n}$ and $\eta \in R^{n}$ were previously defined, $M\in R^{n\times n}$ denotes the inertial matrix (including the added mass), $C\in R^{n\times n}$ is the Coriolis matrix and centripetal forces (including the effects of added mass), $D\in R^{n\times n}$ refers to the damping matrix, $G\in R^{n}$ represents the vector of gravitational forces, and $\tau \in R^{n}$ is the input control vector.

## 3. Ocean Currents

Ocean current is generated by wind, tides, variation of densities and re-circulation of water, among others. The main objective of this work is not to generate a detailed report of this phenomena; nevertheless, it is appropriate to highlight the model of induced ocean currents proposed by Fossen [27]. In the mentioned work, the equations of motion are represented in terms of relative velocity of the vehicle and the currents,

(5) $$\nu_{r}=\nu -\nu_{CI}$$

where $\nu_{CI}={\left[\begin{array}{cccccc} u_{c} & v_{c} & w_{c} & 0 & 0 & 0 \end{array}\right]}^{T}$ is a non-rotational vector of the current velocity according to Equation (3). Note that the linear velocity on the fixed frame can be transformed to linear velocity in the equation by applying the elemental rotation matrices. Let $\left[\begin{array}{ccc} u_{c}^{E} & v_{c}^{E} & w_{c}^{E} \end{array}\right]$ be the current velocity referenced to the Earth-fixed frame. Then the components of the linear velocity on the equation are calculated as follows,

(6) $$\left[\begin{array}{c} u_{c}\\ v_{c}\\ w_{c} \end{array}\right]=J_{1}^{T}\left(\eta_{2}\right)\left[\begin{array}{c} u_{c}^{E}\\ v_{c}^{E}\\ w_{c}^{E} \end{array}\right]$$

Suppose the current velocity in the equation as constant or at least with a minimum variation, so that:

(7) $${\dot{\nu }}_{CI}=0\to {\dot{\nu }}_{r}=\dot{\nu }$$

Then, the relative equations of motion become:

(8) $$M\dot{\nu }+C\left(\nu_{r}\right)\nu_{r}+D\left(\nu_{r}\right)\nu_{r}+G\left(\eta \right)=\tau$$

Now, the current velocity in the Earth-fixed frame $\left[\begin{array}{ccc} u_{c}^{E} & v_{c}^{E} & w_{c}^{E} \end{array}\right]$ can be related to the mean velocity of the current $V_{C}$ through two angles: *α* (angle of attack), *β* (sideslip angle), describing the orientation of $\nu_{CI}$ around the axes *y* and *z* respectively as follows:

(9) $$u_{c}^{E}=V_{C}cos\left(\alpha \right)cos\left(\beta \right)$$

$$v_{c}^{E}=V_{C}sin\left(\beta \right)$$

$$w_{c}^{E}=V_{C}sin\left(\alpha \right)cos\left(\beta \right)$$

where $V_{c}$ is the average currents velocity in the earth-fixed reference frame.

## 4. Self-Tuning Neural Network for PID Control

The tuning of PID (Proportional + Integral + Derivative) controllers depends on adjusting its parameters (i.e., $K_{p}$; $K_{i}$; $K_{d}$), so that the performance of the system under control becomes robust and accurate according to the established performance criteria. The proposed auto-tuning algorithm is based on NN which exhibit the following characteristics:

1. Parallelism and generalization. A NN are able to produce useful outputs for inputs not provided under the learning phase.
2. Non-linearity. A NN can be linear or not allowing it to represent systems generated by nonlinear guidelines.
3. Adaptability. NN are capable of re-adjusting weights and adapting to new environmental situations. This is specially useful when the system offers non-stationary data, that is, the properties involved by the system vary over time.
4. Fault tolerance. When an operational failure occurs on a local part of the network, it lightly affects the global performance.

This property is because of the distributive nature of stored data processed along the neural network.

Consistent with above, this work is based on a backpropagation neural network, which also meets the desired characteristics to accomplish the goal tasks. Recurrent networks with supervised learning structured with delay are widely used in underwater vehicles as mentioned in [17,18], as well as for linear systems with large uncertainties in their surrounding environment as shown in [5,29,30,31].

### 4.1. Control Law

In the discrete time domain, the digital PID algorithm can be expressed as follows [17]:

(10) $$\tau \left(n\right)=\tau \left(n-1\right)+K_{p}\left(e\left(n\right)-e\left(n-1\right)\right)+K_{i}e\left(n\right)+K_{d}\left(e\left(n\right)-2e\left(n-1\right)+e\left(n-2\right)\right)$$

where τ(n) is the original control signal, $e\left(n\right)=\eta_{d}-\eta$ represents the position tracking error, $\eta_{d}$ denotes the desired trajectory, $K_{p}$ is the proportional gain, $K_{i}$ the integral gain, $K_{d}$ the derivative gain, and *n* the sample time.

**Figure 2:**
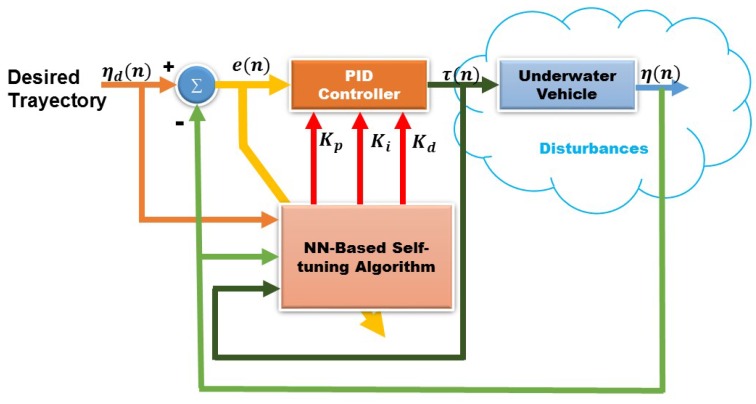
Block diagram of an auto-tuned PID with artificial NN control.

A block diagram of the auto-tuning control with artificial neural network (NN) is shown in Figure 2.

### 4.2. Algorithm Auto-Tuner

**Figure 3:**
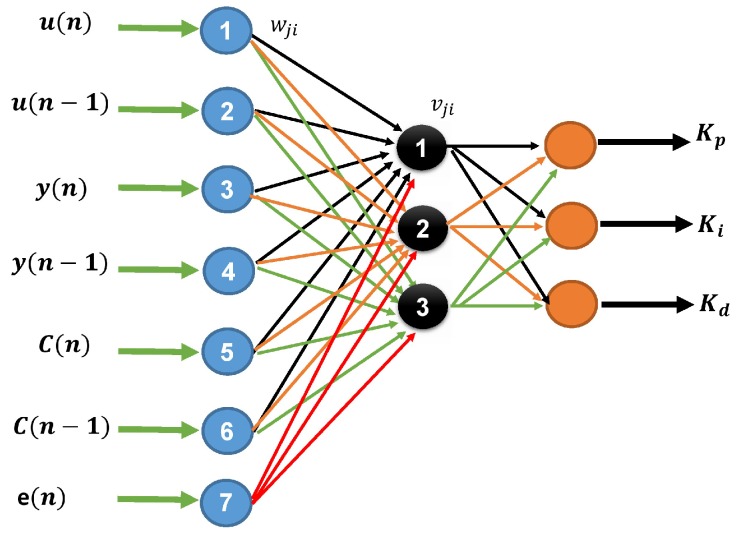
Block diagram of the implemented backpropagation NN.

The algorithm used as auto-tuning is the backpropagation method, chosen for its ability to adapt to changing environments. Operation begins applying the inputs to the network (see Figure 3), this is propagated from the first layer to the hidden layers in, up to produce an output $(K_{p},K_{i}$ and $K_{d})$. The output signal is compared to the desired output and an error signal is calculated for each of the outputs, this is shown in Figure 2. The error outputs backpropagate, starting from the output layer, to all neurons in the hidden layer that contribute directly to the output; however, the hidden layer neurons receive only a fraction of the total error signal. This process repeats iteratively, layer by layer, until all neurons in the network has received an error signal describing its relative contribution to the total error.

Figure 3 presents the topology of the NN used to auto-tune the PID control gains implemented on the ROV. Its structure shows seven neurons on the input layer, three neurons on the hidden layer, and finally another three neurons on the output layer. The neurons placed on the output layer correspond to the PID gains: $K_{p},K_{i},K_{d}$.

where u(n) and u(n−1) are reference inputs (desired trajectory), y(n) and y(n−1) are reference outputs (real trajectory), C(n) and C(n−1) correspond to the control signals, $w_{ji}$ are the weights of the hidden layer, and $v_{ji}$ are the weights of the output layer.

The back-propagation algorithm looks for the minimum of the error function in weight space using the method of gradient descent [3]. The combination of weights which minimizes the error function is considered to be a solution of the learning problem. The activation functions for back-propagation networks is the sigmoid, a real function $s_{c}:R\to \left(0,1\right)$ defined by the expression

(11) $$h_{j}=\frac{1}{1+e^{-S_{j}}}$$

The output of the *j* hidden layer neuron may be calculated by means of:

(12) $$S_{j}=\sum_{i=1}^{3}w_{ji}x_{i}$$

The shape of the sigmoid changes according to the value of $h_{j}$. At the same time, the output layer neuron value will be:

(13) $$u\left(n\right)=\frac{1}{1+e^{-r}}$$

where:

(14) $$r=\sum_{j=1}^{3}v_{j}h_{j}$$

The criteria used to minimize the error correspond to Rojas et al. [32], as:

(15) $$E\left(n\right)=\frac{1}{2}\sum_{k=1}^{t}e_{y}{\left(k\right)}^{2}$$

where $e_{y}\left(k\right)=y_{r}\left(n\right)-y\left(n\right)$.

The minimization procedure consists, as it is known, in a movement in the negative gradient direction of the function E(n) with respect to the weighting coefficients $v_{ji}$ and $w_{ji}$.

The E(n) gradient is a multi-dimensional vector [3] whose components are the partial derivatives $\frac{\partial E(n)}{\partial v_{ji}}$, $\frac{\partial E(n)}{\partial w_{ji}}$.

The weighting coefficients of the input layer are

(16) $$\frac{\partial E(n)}{\partial v_{ji}}=\delta^{1}h_{j}\frac{\partial e_{y}}{\partial e_{u}}$$

The weighting coefficient of the hidden layer are

(17) $$\frac{\partial E(n)}{\partial w_{ji}}=-\delta_{j}^{2}x_{i}\frac{\partial e_{y}}{\partial e_{u}}$$

Using Equations (16) and (17), the adjustments of weighting coefficients $v_{ji}$ (Equtation (18)), $w_{ji}$ (Equtation (19)) can be made by means of the expressions:

(18) $$\begin{array}{ccc} v_{ji}\left(n+1\right) & = & v_{ji}\left(n\right)+\left(a\frac{\partial e_{y}}{\partial e_{u}}\right)\delta^{1}h_{j} \end{array}$$

(19) $$\begin{array}{ccc} w_{ji}\left(n+1\right) & = & w_{ji}\left(n\right)+\left(a\frac{\partial e_{y}}{\partial e_{u}}\right)\delta_{j}^{2}x_{j} \end{array}$$

where *a* is the learning coefficient, $w_{ji}\left(n+1\right)$ is a vector of weights for the hidden layer, $v_{ji}\left(n+1\right)$ is the vector of weights of the output layer and equivalent gain $\frac{\partial e_{y}}{\partial e_{u}}$ is unknown.

## 5. Simulation Results

The auto-tuned PID was evaluated using Matlab/Simulink software. The ODE 45 with a variable step was used, setting the maximum sample step as 0.01 s. The first proposed task consists of moving the robot in a straight line from its start position to a set point, letting x,y,z remain constant while *ψ* is varying. The next task is that the robot begins rising in a spiral motion, perturbed by water currents of considerable intensity. The first perturbation takes place in the first 20 s and its magnitude is $V_{c}=1.1$ m/s with orientation of α=0 and β=0. The second perturbation goes from time 20 to 45 s with a magnitude of $V_{c}=1.1$ m/s and an orientation of α=0 and β=π/2, as set in Equation (9).

### 5.1. Underactuated 6 DOF ROV Kaxan

The Kaxan robot hydrodynamic parameters are included [33].

**Figure 4:**
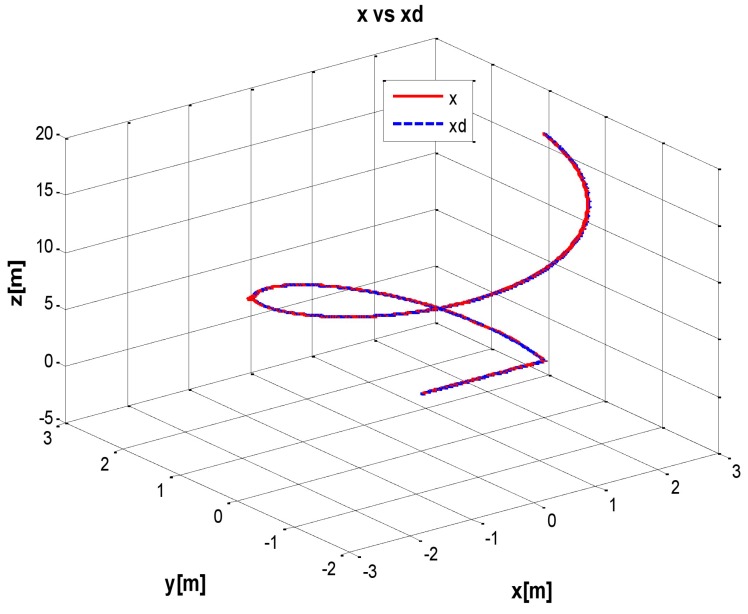
Real trajectory (*X* Solid line) vs. desired trajectory (*X_d_* dotted line), with perturbation.

**Figure 5:**
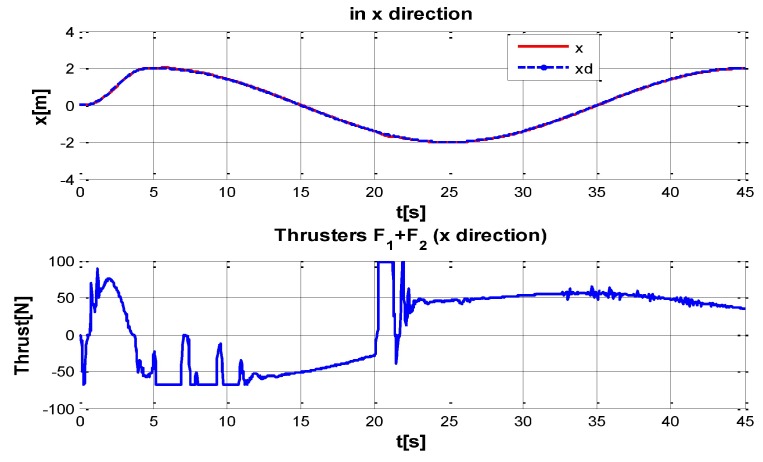
Behavior of the system in x-coordinate with perturbations up and (down) control signal of thruster F1 + F2.

**Figure 6:**
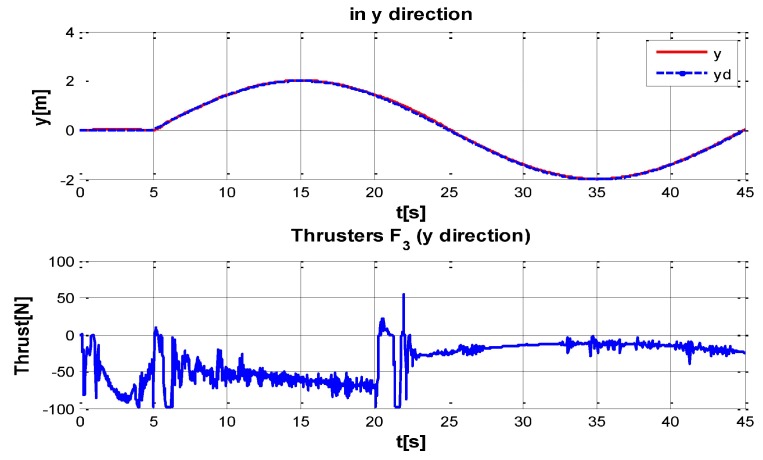
Behavior of the system in y-coordinate with perturbations up and (down) control signal of thruster F3.

**Figure 7:**
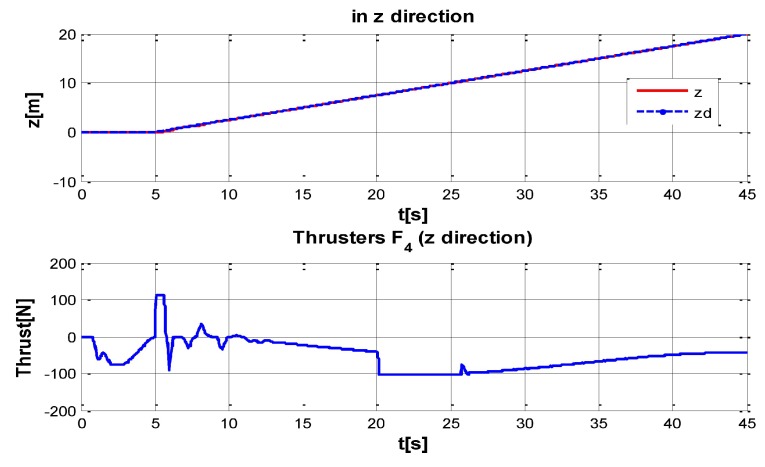
Behavior of the system in z-coordinate with perturbations up and (down) control signal of thruster F4.

**Figure 8:**
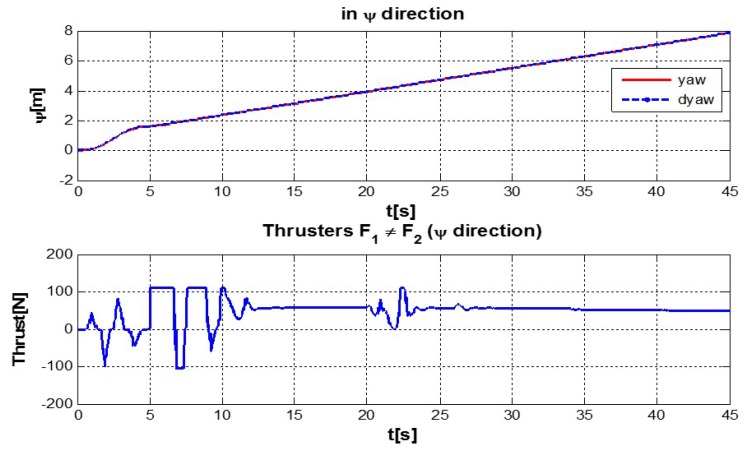
Behavior of the system in *ψ*-coordinate with perturbations up and (down) control signal of thruster *F*1 ≠ *F*2.

The behaviour can be observed in Figure 4, Figure 5, Figure 6, Figure 7 and Figure 8. Figure 4 depicts the trajectory in 3D that the Kaxan robot follows.

Figure 5, Figure 6, Figure 7 and Figure 8, present the interaction of the neural network by modifying the gains from the beginning through time until arriving to a steady state. While the second perturbation is introduced (change of ocean current direction at time 20), neurons detect changes and perform compensation by increasing or decreasing as appropriate, $K_{p},K_{i},K_{d}$ gains.

**Figure 9:**
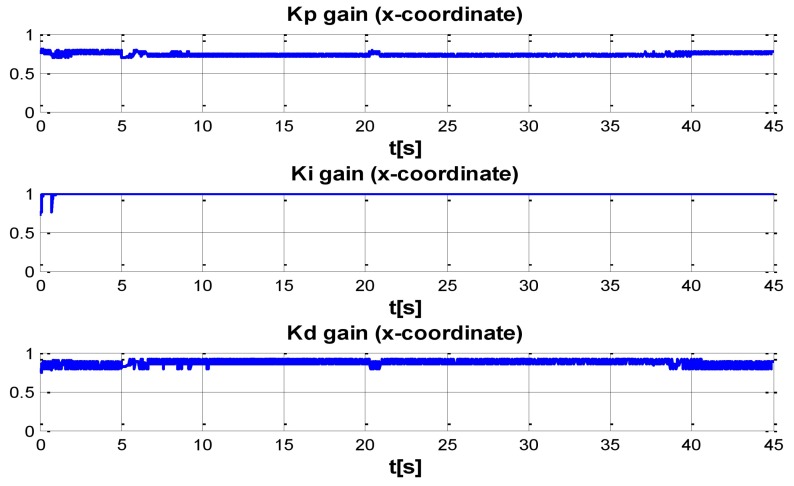
PID gain time behavior in x-coordinate.

**Figure 10:**
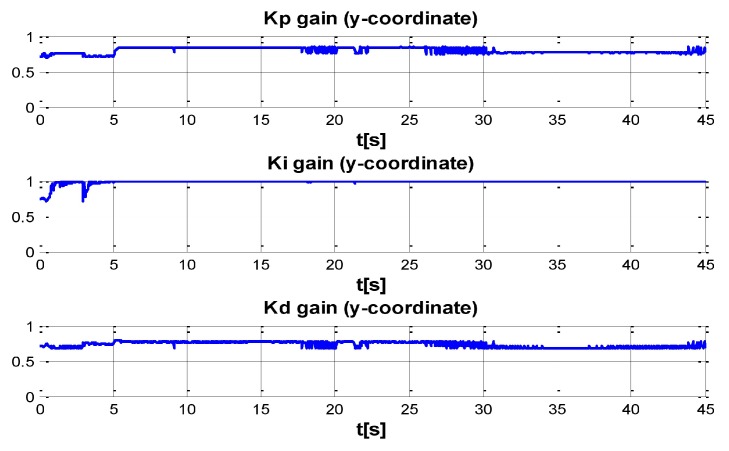
PID gain time behavior in y-coordinate.

**Figure 11:**
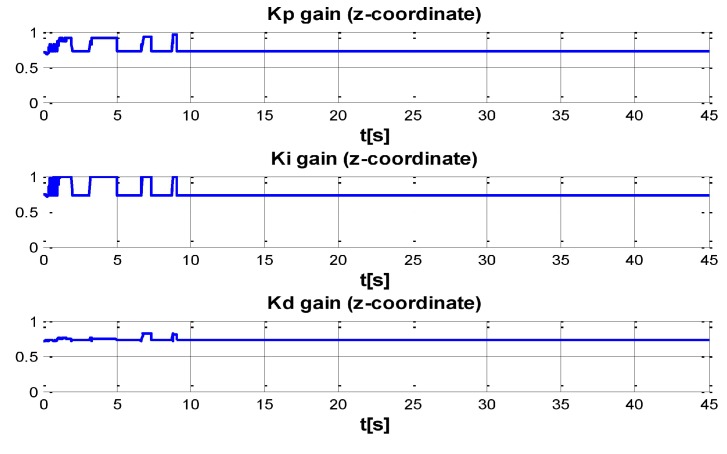
PID gain time behavior in z-coordinate.

**Figure 12:**
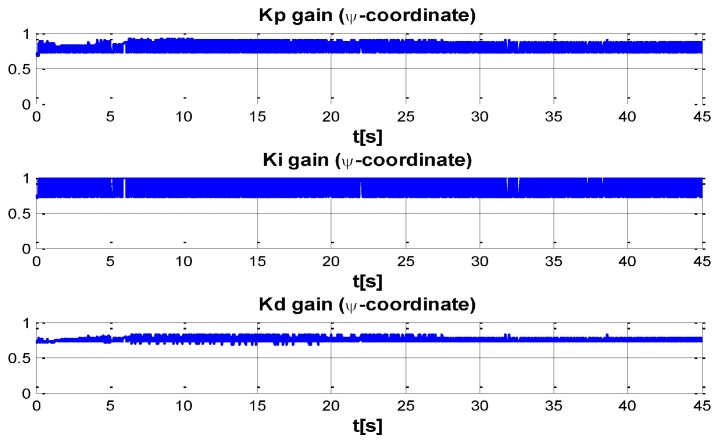
PID gain time behavior in *ψ*-coordinate.

The next set of Figure 9, Figure 10, Figure 11 and Figure 12 indicate the gains $\left(K_{p},K_{i},K_{d}\right)$ obtained by the neural network in every DOF, η=[x;y;z;ψ]. As can be seen, neurons start working from time zero since they absorbed the first perturbation of 20 s in length, and respond to the abrupt change presented when the second perturbation is introduced, thus allowing change of the neural network in order to compensate for the lack of gains in the DOF corresponding to the alterations.

### 5.2. PID vs. Auto-Tuned PID

In order to compare the conventional PID vs. the auto-tuned PID, a statistical indicator was implemented, allowing to determine which one has the best behavior following a trajectory. The mean square error (MSE) lets us estimate the performance of every control by analyzing the error generated in the trajectory tracking.

(20) $$MSE_{R}=\sqrt{MSE_{X}+MSE_{Y}+MSE_{Z}+MSE_{\psi }}$$

where $MSE_{x}$ is the MSE in *x*, $MSE_{y}$ is the MSE in *y*, and so on.

**Figure 13:**
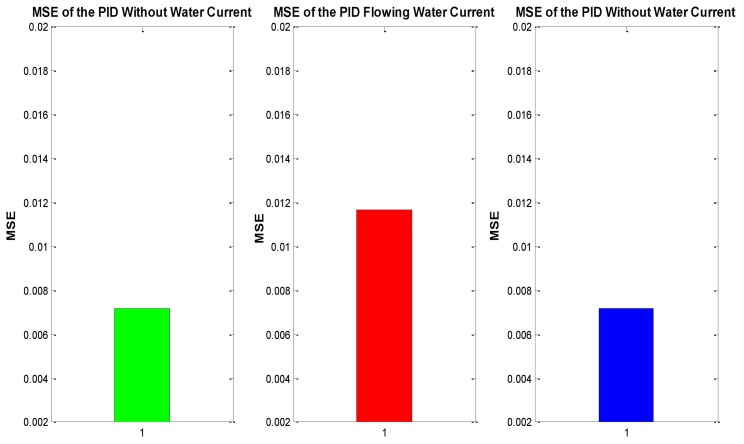
MSE of the conventional PID Controller position tracking trajectory.

**Figure 14:**
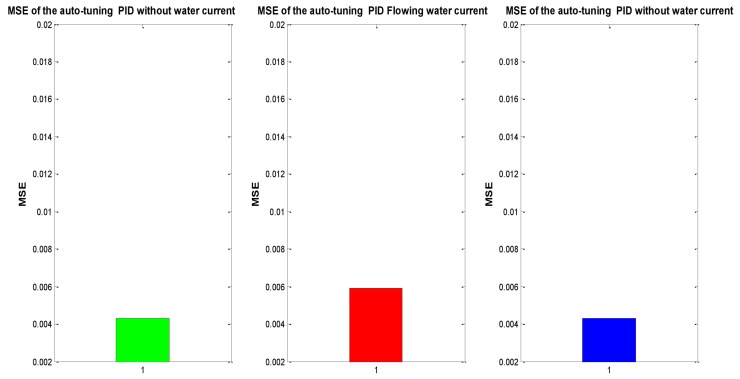
MSE of the auto-tuned PID position tracking.

As mentioned previously, MSE was used to evaluate the tracking performance. Figure 13 shows the evaluation of the experiment mentioned above, which considers the experiment under initial conditions (without water currents) in a 0 s to 45 s time frame in which, afterwards the ocean current appears from time 45 s to 90 s. Finally, at time 90 s the ocean current stops, with discrete time for the PID control being implemented throughout. Figure 14 demonstrates the same experiment with the auto-tuned PID control.

Figure 13 describes the increase of MSE of the conventional PID when perturbation occurs (red bar), while green and blue bars, corresponding to the absence of perturbations, remain steady. Moreover, the MSE of the auto-tuned PID [14] is around a 50% less than the one of the conventional PID, due to the self-tuning algorithm with a NN. Additionally, when perturbation happens, the increase and decrease of the auto-tune MSE is minimum. For this reasons, it is feasible to conclude that the auto-tuned PID has better performance facing changes on the hydrodynamic parameters and perturbations of the surrounding environment.

## 6. Experimental Set up

In this section, the experimental set up as well as the results are discussed. Two sets of experiments are presented, one considering the conventional PID controller and the other one considering the auto-tuned PID proposed in this paper. Both controllers were tested under the same conditions in order to evaluate their performance under disturbances. A comparative analysis in terms of position tracking and energy consumption is given.

**Figure 15:**
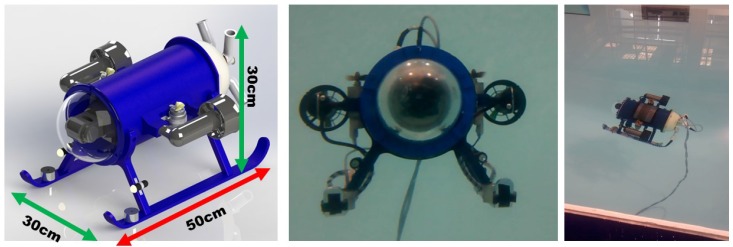
Underactuated mini-ROV under water.

The proposed intelligent control was implemented on an underactuated mini-ROV. This vehicle is a ROV developed in CIDESI named Nu’ukul Ja (which in the Mayan language means ”water instrument”). Its dimensions are: 50 cm long, 30 cm wide, and 30 cm height; as shown un Figure 15. It has a cylindrical pressure chamber of 15 cm diameter where the major part of the electronic architecture is placed. The total weight of the ROV is 10 kg. According to the experimental environment, it was placed in a pool of 2.5 m where both PID controllers (auto-tuned and conventional) were implemented.

**Figure 16:**
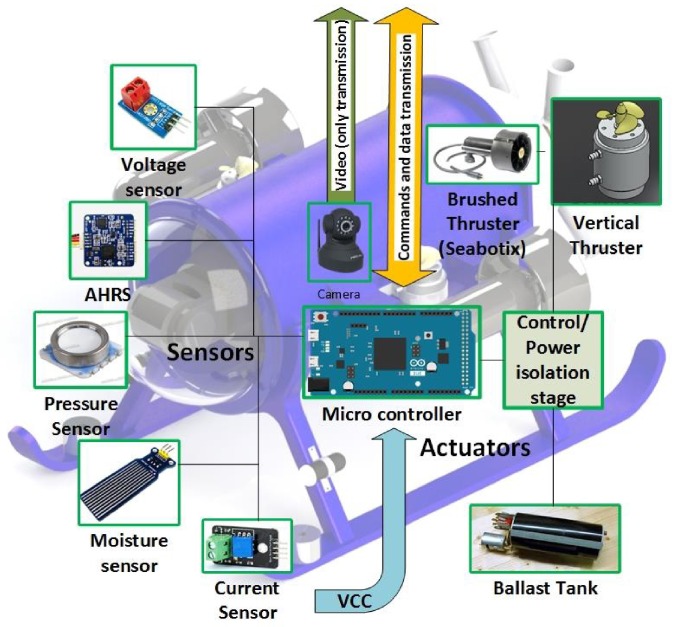
Electronic Architecture of the mini-ROV.

The electronic architecture of the ROV (Figure 16) consist on three groups: instrumentation, signal and data acquisition, and actuators. The instrumentation involves: pressure sensor, leakage sensors, AHRS (Attitude and Heading Reference System), voltage and current sensors. While the signal and data acquisition implicates a micro controller embedded in a development board. Finally, the actuators consist on 4 thrusters used to provide direction and displacement to the vehicle, and an IP camera for inspection missions.

The ROV is connected to the surface by navel string of thirteen wires. Where eight of them are used to receive video from the IP camera, three for the power connections (12 V_*DC*_, 20 V_*DC*_ and Ground), and two for data UART transmission (TX)/reception (RX).

### 6.1. Instrumentation

**Figure 17:**
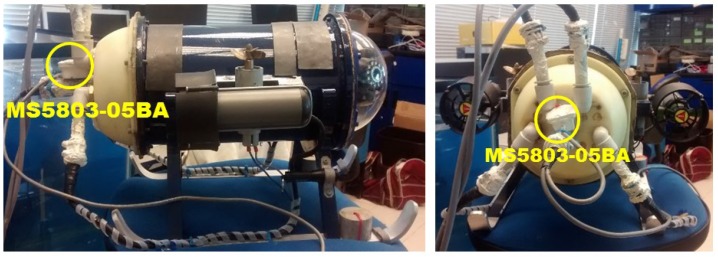
Location Sensor MS5803-14BA.

This ROV has a MS5803−05BA pressure sensor which is placed outside the chamber of the submarine, Figure 17. The sensor is a high resolution barometer which obtain data of the surrounding hydrostatic pressure, acquiring frequencies up to 50 kHz by I2C protocol. Once the hydrostatic pressure is obtained the depth level is calculated by: $h=\frac{P-P_{0}}{\rho g}$, where *h* = depth (m), *P* = hydrostatic pressure (bar), $P_{0}$= atmospheric pressure (bar), *ρ* = water density (kg/m^3^).

In order to sense 3 DOF’s of the ROV (pitch, yaw and roll), the *AHRS UM*7 is a CHRobotics device is used. The AHRS sends NMEA serial packages with a frequency up to 100 Hz. To prevent malfunction of the electronics due to water presence, printed electrodes connected to the controller represent the leakage sensors. Also, 4282 voltage sensor (5 to 1 V_*DC*_ divider) offers an analogical signal of the batteries voltage. *Pololu’s AC*715, a Hall Effect current sensor, allows to monitor the operation of the thrusters.

### 6.2. Signal and Data Acquisition

The ROV has a SAM3X8E *ARM* Cortex embedded in the Arduino Due’s developing board. It has 54 general purpose inputs and outputs, 12 of them PWMs, 12 analog inputs, 4 UART ports and one I2C bus. This controller is used to manage communication between the user and the mini-ROV.

### 6.3. Actuators

**Figure 18:**
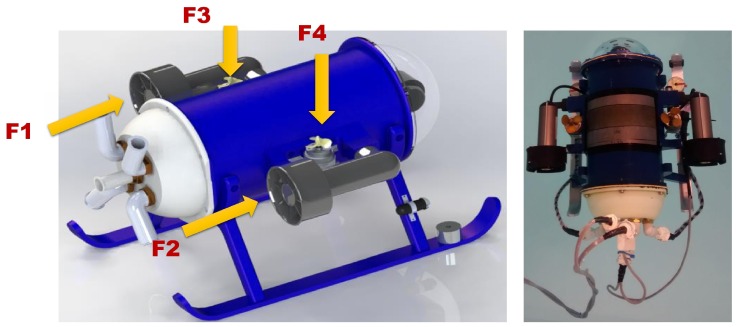
Thruster location.

As mentioned above, to displace and lead the submarine in one plane, two brushed *SeaBotix* BTD150 thrusters are placed horizontally on each side of the underwater robot. These thrusters are powered by 20 V_*DC*_@ 4 A. To dive the submarine, 2 brushed thrusters were placed vertically on each side of the ROV (beside the lateral thrusters, Figure 18); these are basically modified fuel pumps with 4 cm propellers attached to their shaft. Each brushed motor consume 12 V_*DC*_@1.5 A .

### 6.4. Results

**Figure 19:**
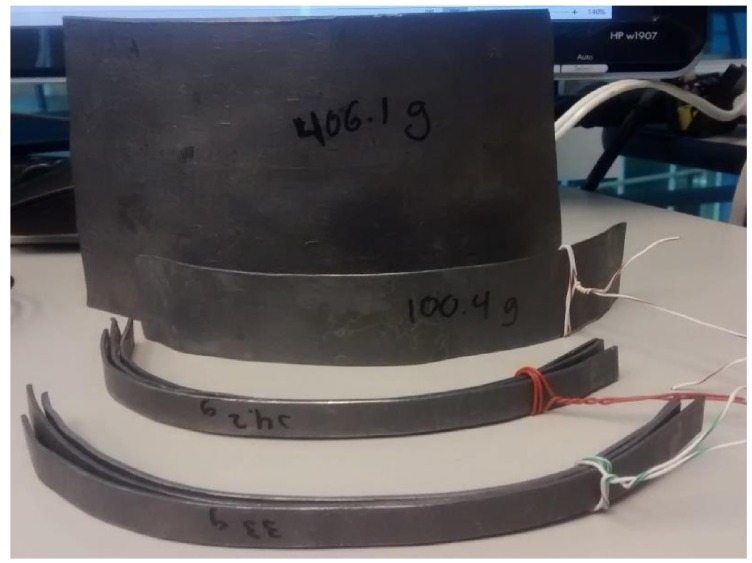
Weight (disturbance).

The controls were evaluated by performing a data capture of 3 m, once the ROV was placed 1m underwater. In the first minute non disturbance took place. After this time, the weight was increased by 400 g (Figure 19) until a two minute mark.

The gains of the conventional PID control were obtained by means of the NN. The ROV was requested to get the set point of 1 m depth by using the Auto-tuned PID controller. Once the ROV reached the stability and the PID gains, computed by the NN, became stationary, these gains were programmed into the conventional PID as Kp, Kd, and Ki. This the way the conventional PID gains were tuned. It is important to remark that once the conventional PID was tuned, the gains remained constant along the experiment, even when the disturbances took place, unlike the Auto-tuned PID controller wherein gains were dynamically changing to attain the better performance along the experiment.

**Figure 20:**
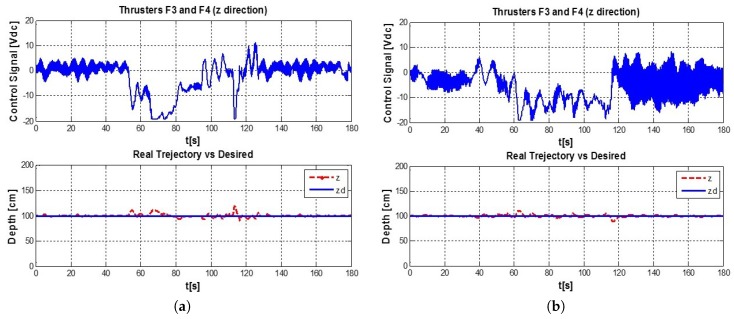
(**a**) Conventional PID Controller, (**b**) auto-tuned PID.

Finally, in the last minute of data capture the weight was removed. Figure 20 shows the desired trajectory (Solid line) vs. the real path (dotted line), also the control signals for thrusters F1 and F2 are displayed.

Apparently, the control signal given by the auto-tuned PID (shown in Figure 20), seems to be more active than the conventional PID’s signal; though, the root mean square (RMS) value of each one (the complete experiment), shows that the RMS of the conventional PID is 6.8874 whereas in the auto-tuned PID is 6.6781. The auto-tuned PID has a 3.038% Of energy saving against the conventional PID.

**Figure 21:**
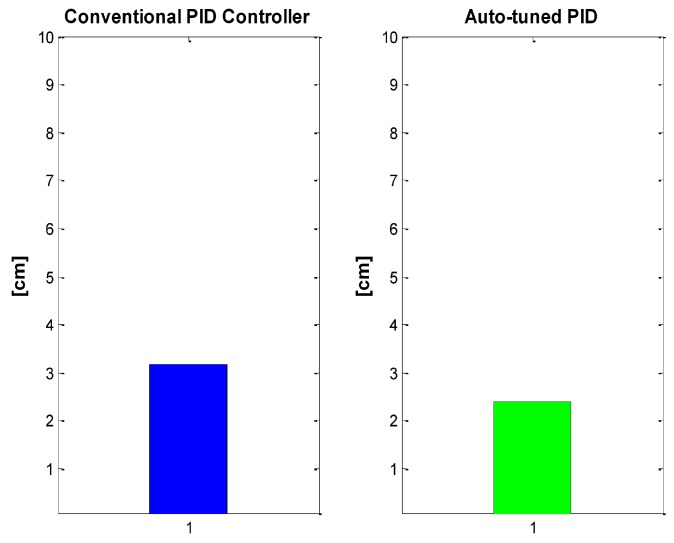
MSE: Conventional PID Controller (**left**) vs. auto-tuned PID (**right**).

MSE offers a better notion of the results, leading to the conclusion that the neuronal PID is better than PID fixed gains, as can be seen in the Figure 21.

**Figure 22:**
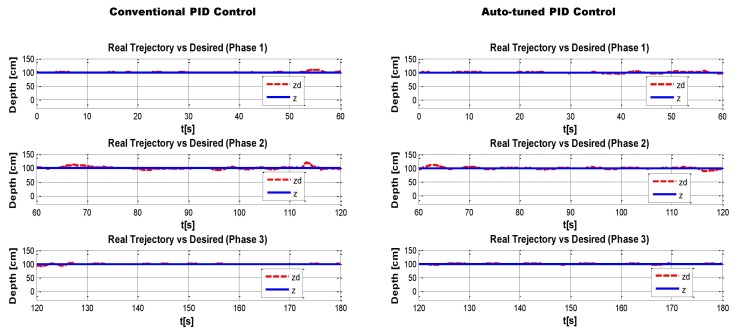
Conventional PID Controller vs auto-tune PID, segmented in 3 phases of 1 m each.

**Figure 23:**
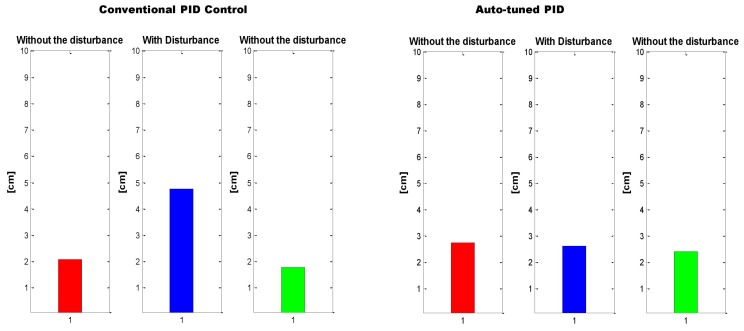
MSE: Conventional PID Controller (**left**) vs. auto-tune PID (**right**).

In order to determine where the neural PID has a better performance, the test was divided in three phases corresponding to: where no disturbance took place, when the disturbance is added and again when the disturbance stopped, as can be seen in Figure 22. Once again, to compare the auto-tuned PID vs. the conventional PID controller with fixed gains, the MSE of every phase was obtained and it is shown in Figure 23.

## 7. Conclusions

The actual work presents the development of a control algorithm to automatically tune the gains of a PID control, based on a neural network. The control algorithm was implemented on ROVs for trajectory tracking with unknown disturbances. The algorithm performance was evaluated in two instances: a numerical simulation and implemented on a ROV in real-time. The numerical simulation took place with the non-linear hydrodynamics of ROV Kaxan with 4 of the 6 DOF actuated; including disturbances of ocean currents in different directions. In reference of the second validation, it was implemented in a mini-ROV for the depth DOF, in order to validate in real-time the auto-tuned PID control. A comparative study between the conventional PID and the auto-tuned PID (proposed here) was discussed. The study took into consideration two criterions to assess the performance of each controller: position tracking error and energy consumption, leading to the conclusion that the proposed controller attained the best performance with less energy.
